# Effects of Astaxanthin on Chronic Exercise Fatigue

**DOI:** 10.33549/physiolres.935603

**Published:** 2025-08-01

**Authors:** Shan LIU, Klára DAĎOVÁ

**Affiliations:** 1 Faculty of Physical Education and Sport, Charles University, Prague, Czech Republic; 2 GuiYang Healthcare Vocational University, Guizhou, China; 3 Research Institute of Sports and Health, Tianjin University of Sport, Tianjin, China

**Keywords:** Carotenoid, Exercise recovery, Mitochondria, Antioxidant Capacity

## Abstract

Astaxanthin is a natural, small-molecule compound with anti-inflammatory and antioxidant properties that has broad potential for use in alleviating exercise fatigue. This study investigated whether astaxanthin can attenuate the onset of fatigue, prolong the time to exhaustion, and enhance post-exercise recovery using a rat model of chronic exercise fatigue. Twenty male rats were trained for 8 weeks to establish the chronic exercise fatigue model. During training, 10 rats were randomly assigned to receive astaxanthin intragastrically and 10 rats received soybean oil alone. After the intervention, 5 rats from each group were divided into astaxanthin (AX) and control groups. The remaining rats were divided into astaxanthin-exercise (AXE) and exercise control groups, respectively, and underwent exhaustive exercise. Astaxanthin alleviated chronic exercise fatigue by improving antioxidant capacity (↑CAT, GSH-Px, GSH/GSSG; p<0.05–0.01) and mitochondrial function (↑MMP, ST3/ST4; p<0.01–0.001). It prolonged exercise endurance (↑time to exhaustion; p<0.001), reduced muscle damage (↓BUN, CK; p<0.01) and accelerated recovery (↑Liver glycogen, NEFA; p<0.001). Astaxanthin appears to improve skeletal muscle antioxidant capacity and mitochondrial function in chronic exercise fatigue in rats, providing a theoretical basis for fatigue management in exercise training.

## Introduction

Exercise fatigue is the inability of the body to maintain a given level of function and/or a given exercise intensity [1]. It is a normal physiological phenomenon and a warning signal in human exercise, forcing the body to reduce the exercise load to prevent failure [2]. However, during long-term exercise, fatigue cannot be eliminated in time. It builds up in the body, leading to chronic exercise fatigue [3]. Therefore, it is imperative for athletes seeking to improve their performance to identify safe and effective strategies to delay the onset of fatigue and accelerate recovery.

Astaxanthin (chemical name: 3,3′-dihydroxy-4,4′-diketone-β, β′-carotene and molecular formula: C_40_H_52_0_4_) is an unsaturated terpene and an ideal antioxidant that has been extensively studied recently [4–6]. One study showed that mice given astaxanthin by gavage for 5 weeks had a significantly longer time to exhaustion in swimming compared to the control group, indicating that astaxanthin increases muscle strength and endurance and improves lipid metabolism during exercise, with potential glycogen retention [7]. The effects of dietary supplementation with astaxanthin on exhaustive exercise-induced injury in C57BL/6 mice were investigated and showed that astaxanthin reduced oxidative damage, protected cardiac and gastrocnemius muscles and reduced myeloperoxidase production [8]. Previous studies have also reported that in astaxanthin-supplemented mice, after swimming exercise, blood lactic acid concentration significantly decreased, plasma free fatty acid levels increased, blood glucose consumption decreased, and the time to exhaustion became longer, indicating that their exercise capacity was significantly improved [9–10]. In addition, recent evidence suggests that astaxanthin inhibits glutathione peroxidase (GSH-Px) and catalase (CAT) activity in plasma or muscle of moderately trained mice; downregulates transcription of Nrf2 and Nrf2-dependent enzymes in skeletal muscle or heart; reduces malondialdehyde levels in myocardiocytes, plasma and muscle; and reduces oxidative stress damage [11]. Taken together, these data suggest that astaxanthin has a protective effect against exercise injury. Nuclear magnetic resonance metabolism histology studies found that astaxanthin supplementation improved the body’s antioxidant and exercise capacity during acute exercise which was associated with changes in amino acid and lipid metabolism [12]. However, no studies have determined whether astaxanthin also plays a role in the development and recovery of chronic exercise fatigue.

Therefore, using a rat model of chronic exercise fatigue, we investigated the effects of astaxanthin on the development and resolution of long-term chronic exercise fatigue and explored the potential mechanisms to provide a theoretical basis for fatigue management in exercise training.

## Materials and Methods

### Animals

Twenty 8-week-old healthy male Sprague-Dawley rats (200 ± 20 g each) were purchased from Beijing Weitong Lihua Experimental Animal Technology Co. (China). All experimental animals were of specific pathogen-free grade, with no genetic modifications. They were housed in cages and fed standard chow (protein ≥ 18 %, fat ≥ 4 %, and carbohydrate ≥ 4 %; Beijing Weitong Lihua Experimental Animal Technology Co., Ltd.). The main components of this diet were soybean meal, flour, bran, rice minerals and trace elements. The housing conditions for the rats were: room temperature of 20 °C–26 °C, 12-h light/dark cycle, and relative humidity of 40–70 %, with ad libitum access to food and drinking water.

The ethics governing the use and conduct of experiments on animals were strictly adhered to, all experimental protocols were approved by the Medical Research Ethics Committee of Tianjin University of Sport, and all experiments were performed in accordance with relevant guidelines and regulations.

### Reagents

Astaxanthin (AstaREAL ® rainy red algae powder, AstaReal AB, Sweden) was provided free of charge by Tianjin Baillie Biotech Co., Ltd. (China). Creatine kinase (CK) active test kit, hydrogen peroxide enzyme (CAT) active test kit and glutathione peroxidase (GPX) active test kit were provided by Beijing Solebao Technology Co., Ltd. (China). The glycogen measurement kit, prototype glutathione (GSH) test box and oxidized glutathione (GSSG) content test box were purchased from Suzhou Keming Biotechnology Co., Ltd. (China). The non-esterified fatty acid (NEFA) test kit was purchased from Nanjing Built Bioengineering Research Institute (China). The Reactive Oxygen Species (ROS) assay kit were provided by Beijing Solebao Technology Co., Ltd. (China). Other reagents were purified for use in the analyses.

### Animal grouping and intervention

**Fig. 1:**
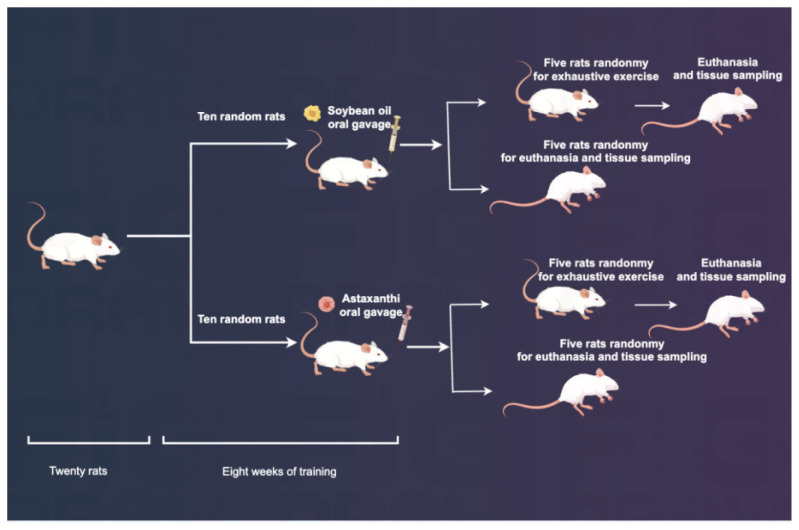
Experimental Workflow

Twenty rats were trained for 8 weeks to establish the chronic exercise fatigue model as described by Liu [13], details are in Table 1. The training protocol consisted of progressive sessions five days per week for eight weeks, starting at a speed of 10 m/min (weeks 1–2), gradually increasing to 30 m/min (weeks 7–8), with the incline rising from 0° to 5°. Each session duration increased progressively from 20 to 40 minutes. During training, 10 randomly selected rats received astaxanthin intragastrically (3 mg/100 g body weight/day, diluted in soybean oil) and another 10 rats received an equal amount of soybean oil alone. Immediately after the inter-vention, 5 rats from each group were randomly selected for measurements of fatigue-related parameters. They were divided into astaxanthin (AX) and control (Con) groups. The remaining 10 animals underwent exhaustive exercise 24 h after the intervention and were divided into the astaxanthin exhaustive exercise (AXE) and exhaustive exercise control (E) groups, respectively. Exhaustive exercise was performed on a treadmill with a 0° incline and a speed of 20 m/min. The animal was considered exhausted if it could not maintain the initial running speed, did not respond to stimulation, stopped after signs of shortness of breath, looked tired, and had a slow response to stimulation at the end of the exercise. The specific grouping is shown in the Appendix (Table 2). The intervention procedure is shown in Figure 1.

### Material collection

Rats were anesthetized with 3 % pentobarbital sodium (1 ml/kg) by intraperitoneal injection 24 h after the last exercise or fatiguing session. From whole blood, 3 ml was collected from the apex of the heart and kept at room temperature for 20 min. The blood samples were then centrifuged at 3500 rpm for 15 min at 4 °C using a high-speed centrifuge (Beckman Coulter, USA) to obtain the serum. After the animals were euthanized, the liver and hindlimb gastrocnemius muscles were harvested. An adequate amount of gastrocnemius muscle was used for immediate extraction of mitochondria. The liver was quickly placed in liquid nitrogen and stored at −80 °C for subsequent molecular biology experiments.

### Determination of fatigue index

After centrifugation and extraction of animal serum, blood urea nitrogen (BUN), NEFA concentration and CK activity were measured using the respective kits according to the manufacturer’s instructions. The liver was extracted and its glycogen content was measured according to the kit instructions.

### Determination of oxidation index

After centrifugation and extraction of animal serum, CAT, GSH-Px, GSH and GSSG activities were measured according to the kit instructions and the GSH/GSSG ratio was calculated.

### Mitochondrial function assay

Rat gastrocnemius muscles were placed in a clean glass dish on ice. A small amount of mitochondrial extraction medium was added, then the muscles were dissected and transferred to a homogenization tube. After adding an appropriate amount of mitochondrial extraction medium, the samples were slowly homogenized several times in an ice bath and then transferred to a centrifuge tube. The mitochondrial extraction medium was added to the homogenization tube and the tissues were homogenized again until the tissue block became a white precipitate with no obvious color. The homogenates were then centrifuged at 4 °C and 1000 g for 10 min and the supernatant was removed. The samples were then centrifuged again at 4 °C and 10000 g for 10 min and the supernatant was removed. Subsequently, 450 μL of mitochondrial preservation medium was added to the samples and mixed well to obtain crude mitochondria. Mitochondrial membrane potential (MMP) was measured using a 20/20n luminometer (Turner Biosystems Instruments, USA). Mitochondrial function, mitochondrial state III (ST3) and state IV (ST4) respiratory functions were measured using the Oxygraph-2K high-resolution cellular respirometer (OROBOROS, Austria), and the respiratory control ratio (ST3/ST4) was calculated.

### Statistical analysis

The data obtained were expressed as mean ± standard deviation and processed using SPSS statistical analysis software (version 23.0). Comparisons between groups: independent t-test or Mann-Whitney U test (according to data normality) for two groups. Statistical significance was defined as p<0.05.

## Results

### Anti-fatigue effect of astaxanthin on chronic exercise fatigue

#### Anti-fatigue capacity

**Fig. 2:**
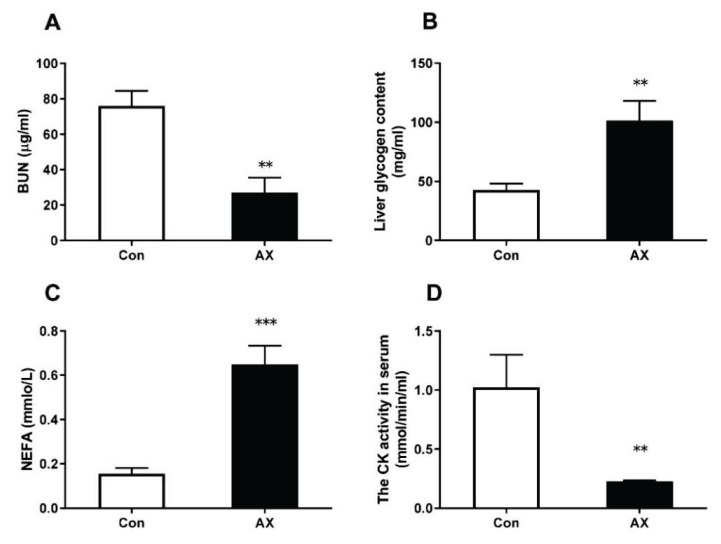
Changes in anti-fatigue indexes in rats with chronic exercise fatigue. ** p<0.01, *** p<0.001; Con, control; AX, astaxanthin

Exercise duration of astaxanthin-treated rats was longer than that of untreated rats (*p*<0.001). In rats with chronic exercise-induced fatigue, astaxanthin-treated rats exhibited lower serum BUN content (p<0.01) and CK activity (p<0.01) and higher liver glycogen (p<0.01) and serum NEFA (p<0.001) levels compared to the Con group (Fig. 2).

#### Antioxidant capacity

**Fig. 3:**
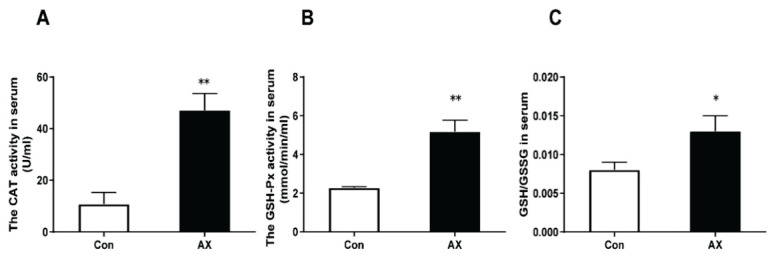
Changes in antioxidant indexes in rats with chronic exercise fatigue. * p<0.05, ** p<0.01; Con, control; AX, astaxanthin

Figure 3 shows the changes in the antioxidant capacity of skeletal muscles in rats after chronic exercise fatigue. Compared with the Con group, the serum CAT (*p*<0.01) and GSH-Px (*p*<0.01) activities and GSH/GSSG ratio (*p*<0.05) were significantly increased in the AX group.

#### Mitochondrial respiratory function

**Fig. 4:**
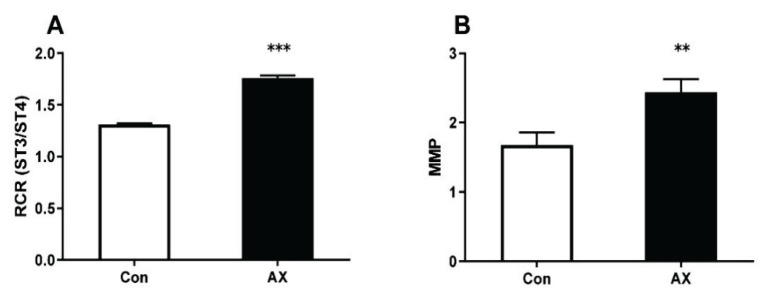
Changes in mitochondrial function in rats with chronic exercise fatigue. ** p<0.01, *** p<0.001; Con, control; AX, astaxanthin

As demonstrated in Figure 4, the mitochondrial respiratory rate and MMP of rats in the AX group were significantly higher than those of rats in the Con group (*p*<0.001 and 0.01, respectively).

### Role of astaxanthin in promoting recovery from exhaustive exercise after chronic exercise fatigue

#### Fatigue-relieving energy level

**Fig. 5:**
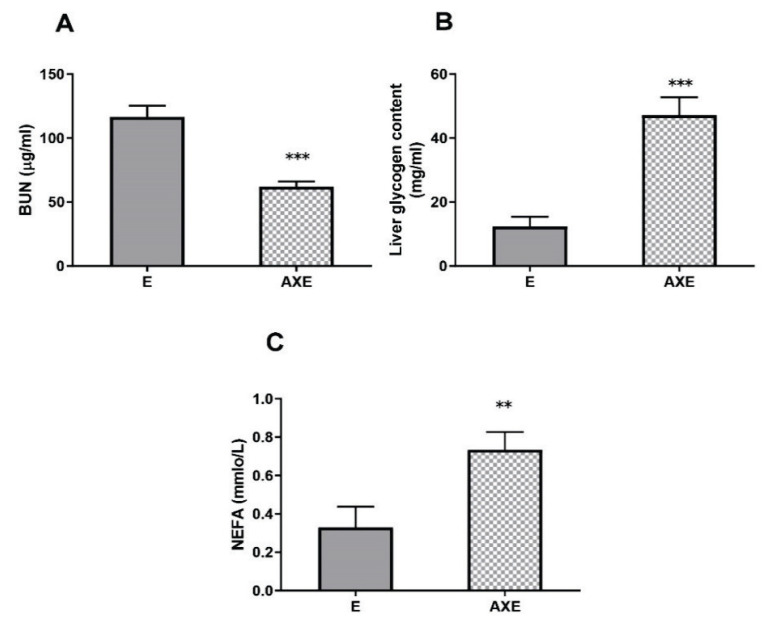
Changes in fatigue recovery in rats that underwent exhaustive exercise ** *p*<0.01, *** *p*<0.001; E, exhaustion exercise control; AXE, astaxanthin exhaustion exercise.

Figure 5 describes the values of rats following chronic exercise fatigue and a single cycle of exhaustive exercise, with a subsequent 24-hour period of rest. Compared with the E group, the serum BUN content was significantly decreased (*p*<0.001), and liver glycogen (*p*<0.001) and serum NEFA levels were significantly increased (*p*<0.001) in the AXE group.

#### Effects on antioxidant capacity

**Fig. 6:**
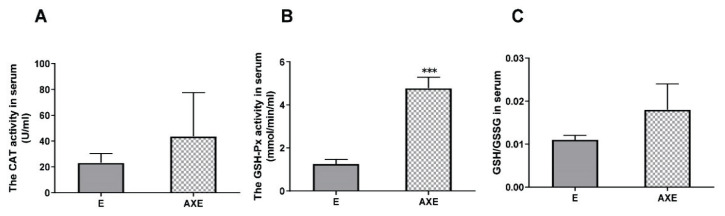
Changes in antioxidant indexes in rats that underwent exhaustive exercise. *** p<0.001; E, exhaustion exercise control; AXE, astaxanthin exhaustion exercise.

Similarly, serum GSH-Px activity was found to be significantly elevated in the AXE group in comparison to the E group (p<0.001). However, no statistically significant differences were observed in CAT activity and the GSH/GSSG ratio, although an increasing trend was noted (Fig. 6).

## Discussion

Exercise fatigue is a prevalent issue in both competitive sports and fitness regimes. The utilization of exogenous supplements, such as astaxanthin, renowned for its robust antioxidant properties, to alleviate exercise fatigue is gaining popularity. This study investigates astaxanthin’s function in chronic exercise fatigue to provide insights for sports medicine and the management of chronic fatigue. Additionally, it seeks to contribute to the development of fatigue intervention strategies and dietary supplements for chronic fatigue syndrome.

### The anti-fatigue effect

Exhaustive exercise time is a comprehensive performance of the body’s motor function and can reflect not only the body’s ability to resist fatigue, but also its ability to resist oxidative stress [4]. It has been reported that astaxanthin intervention prolonged the time to exhaustion in mice during swimming. Furthermore, Campbell [9] found that mice treated with 300 mg/kg/day astaxanthin exhibited significantly improved skeletal muscle strength after 8 weeks of exercise training, as well as a longer time to exhaustion in the treadmill test. Utilizing a chronic exercise fatigue model, we observed that the time to exhaustion of rats was increased after 8 weeks of astaxanthin intervention. These findings demonstrate that astaxanthin enhances both acute and chronic exercise performance, thereby improving endurance during regular training and exhibiting anti-fatigue effects in chronic exercise models. In order to accurately explore the anti-fatigue effect of astaxanthin, the relevant biochemical indexes related to fatigue were tested. In endurance exercise, energy depletion is usually the main factor causing fatigue; therefore, reducing this depletion can further delay fatigue and provide energy.

As the duration of exercise is increased, the consumption of liver glycogen can reach 90 % of maximum capacity, leading to a decrease in blood glucose concentration to the lower limit of the normal range, which is a contributing factor to the onset of exercise fatigue [1]. In the chronic exercise fatigue model utilized in this study, the liver glycogen concentration in the rats treated with astaxanthin was significantly higher than in the control rats. It suggests that the increase in liver glycogen storage contributes to the maintenance of blood glucose homeostasis during exercise. This is consistent with Zhou *et al*. [11] which found that mice supplemented with astaxanthin for 3 weeks had significantly higher blood glucose concentrations after swimming exhaustion exercise compared to controls.

During physical exertion, the energy balance within the organism is subject to disruption, resulting in a decline in muscle glycogen content and blood glucose levels. Concurrently, protein and amino acid catabolism is augmented, and blood urea nitrogen (BUN) levels increase [14]. Serum BUN content has been demonstrated to be associated with the functional state of the body, the extent of fatigue, and the magnitude of exercise-related stress. Following 30 days of gavage with varying doses of astaxanthin and a weight-bearing swimming experiment, the serum BUN content in the high-dose group (150 mg/kg/day) exhibited a significant decrease compared to the control group [15]. In this experiment, following astaxanthin intervention, serum BUN content was significantly reduced in rats with chronic exercise fatigue in comparison to the control group, suggesting that astaxanthin reduces protein consumption during exercise, conserving protein and contributing to the anti-fatigue effect. During exercise, as glucose is continuously consumed, neutral fat stored in the adipose tissue is broken down into free fatty acids for utilization by the body. In a study by Takami *et al*. [16], mice receiving astaxanthin supplementation for 4 weeks exhibited increased plasma NEFA concentrations following exhaustive exercise in comparison to the control group. These long-chain fatty acids are transported to the mitochondria via the carnitine palmitoyltransferase complex (CPT), thereby providing an energy supply.

During periods of strenuous exercise, CPT1 is subject to oxidative damage, which impairs its function in transporting long-chain fatty acids. Conversely, astaxanthin, a highly lipophilic compound, accumulates on the mitochondrial membrane following ingestion [17–18]. This provides a protective effect against oxidative damage to CPT1, thereby enhancing long-chain fatty acid transport and, consequently, indirectly increasing lipid metabolism, providing energy for exercise, and delaying the onset of fatigue. In the present study, astaxanthin intervention in chronic exercise fatigue rats also resulted in a significant increase in serum NEFA concentrations, suggesting that astaxanthin can enhance lipid metabolism during chronic exercise fatigue. During exercise, mechanical stretching stimuli can lead to skeletal muscle cell damage and increased membrane permeability, which causes intracellular CK to escape and be released into the blood, resulting in varying degrees of elevated serum CK activity [19], as a sensitive indicator of muscle injury. In the chronic fatigue rat model employed in this study, the serum CK activity in the AX group was significantly lower than that in the Con group, suggesting that astaxanthin can reduce the level of skeletal muscle injury caused by chronic exercise fatigue, thereby prolonging exercise time and delaying the onset of fatigue.

Wolf *et al*. [20] found that astaxanthin was able to maintain normal MMP and high respiratory rate, and redox state of the mitochondria under oxidative stress conditions even at nanomolar concentrations. Furthermore, the results of the present study demonstrate that astaxanthin can upregulate the expression of adenylate-activated protein kinase α-1, peroxisome proliferator-activated receptor-γ, and CK2 in the mitochondria of myasthenic skeletal muscles and promote mitochondrial biosynthesis [21]. In the present study, the mitochondrial respiration rate of the rat gastrocnemius muscle was significantly enhanced in the AX group compared with the Con group, suggesting that astaxanthin improves the mitochondrial function of skeletal muscles in rats with chronic fatigue, which, in turn, may improve their energy supply and exert an anti-fatigue effect.

The mitochondrial membrane is a pivotal site for the maintenance of oxidative phosphorylation and ATP production in cellular mitochondria. Damage to mitochondrial membranes can be determined by detecting changes in MMP [22]. Furthermore, oxidative phosphorylation in the mitochondrial membrane is one of the pathways through which reactive oxygen species (ROS) are produced, and excess accumulation of ROS results in mitochondrial membrane damage and a decrease in MMP [23]. The present chronic fatigue experiment revealed that MMP was significantly higher in the gastrocnemius muscle of rats in the AX group in comparison to Con group. We hypothesize this might be due to the antioxidant protective effect of astaxanthin, which reduces mitochondrial membrane damage. However, mitochondrial ROS levels were not measured in this experiment. Nevertheless, the results demonstrate that astaxanthin significantly reduced the abnormal alteration of MMP in the gastrocnemius muscle of rats and protected mitochondrial function. The strength of mitochondrial function directly affects the body’s energy supply capacity and the length of exercise. Therefore, the results of our study suggest that astaxanthin can enhance energy supply, have an anti-fatigue effect, and prolong exercise time by improving mitochondrial function.

In 1956, Harman [24] proposed the free radical theory, which posits the existence of free radicals as a standalone entity within normal organisms. These radicals are believed to engage in a series of reactions with various biomolecules present within the body, resulting in the occurrence of damage. They also reported that strenuous exercise leads to an augmentation in the production of these free radicals within the body. Among the various factors contributing to this increase, the researchers identified reactive oxygen species (ROS) as a primary agent responsible for disruptions in muscle redox homeostasis. This, in turn, has the capacity to induce muscle fatigue and consequently affects the exercise capacity of the body as a whole. In conditions of oxidative stress, the body utilizes a combination of endogenous (e.g., superoxide dismutase (SOD), catalase (CAT), and GSH-Px) and non-endogenous (e.g., vitamin C) antioxidants in a synergistic manner to avert ROS-induced damage to tissue cells [25]. Notably, GSH-Px has been shown to catalyze the conversion of GSSG to GSH, with the GSH/GSSG ratio serving as an indicator of the redox state of the organism. In a study by Polotow *et al*. [26], the administration of astaxanthin for a period of 45 days resulted in an augmentation of the GSH-Px antioxidant response in the muscle tissue of flounder during periods of exercise in rats. This was accompanied by a decline in oxidative stress levels and a delayed fatigue onset. In this chronic exercise fatigue model, the serum CAT and GSH-Px activities and GSH/GSSG ratio of rats treated with astaxanthin were significantly higher than those of control rats. Therefore, astaxanthin can enhance the antioxidant capacity of the organism under repeated oxidative stress in chronic exercise fatigue.

### The fatigue recovery promoting effect

In general, recovery can take place both during and after exercise, with post-exercise recovery being more prominent. Poor recovery can lead to long-term fatigue, a decline in sports performance, and sports injuries. Post-exercise energy recovery is critical, and studies have found that supplementation of carbohydrate and protein-related mixtures during exercise recovery in endurance athletes improves their recovery [27]. Exogenous supplementation before and after exercise is now becoming quite common. A hallmark of chronic fatigue syndrome is the profound and protracted nature of fatigue, which often resists recovery following exercise. In the present study, a substantial augmentation in liver glycogen content was observed in the astaxanthin-treated group subsequent to a single bout of exhaustive exercise, thereby signifying that astaxanthin facilitates the restoration of energy substrates following exercise in chronically fatigued rats.

Changes in blood urea levels have also been demonstrated to reflect the recovery of body functions [28] as prolonged exercise has been shown to accelerate the rate of protein metabolism in the body. Following the cessation of exercise, the clearance rate of urea by the body is accelerated to restore body functions, so the level of blood urea metabolism can reflect the body’s ability to recover to a certain extent. In our study, 24 hours after exhaustive exercise, the serum BUN content significantly decreased in the AXE group compared with the E group, indicating that astaxanthin can accelerate the clearance of BUN and promote recovery. The increase in blood NEFA level during exercise is due to enhanced lipid metabolism to maintain the energy supply for muscle contraction. Increased fatty acid utilization during exercise has been shown to reduce the rate of glycogen consumption and improve the performance of endurance exercise [29–30]. It has been shown that athletes who underwent only 7 days of astaxanthin supplementation during endurance exercise had significantly higher rates of body fat oxidation [31]. Following the cessation of muscle contraction, other tissues enhance the utilization of free fatty acids in the blood through oxidation and esterification, which in turn removes excess free fatty acids from the body [32].

In our study, 24 hours after exhaustive exercise, serum NEFA levels were higher in the AXE group than in the E group. We hypothesize this is because astaxanthin was dissolved in oil during gavage, which interferes with the efficiency of free fatty acids being oxidized and esterified after exercise, affecting lipid metabolism; this, in turn, affects serum NEFA during the post-exercise recovery period; however, this needs to be investigated further. Heavy exercise leads to disruption of the mitochondrial structure and respiratory dysfunction; further, it induces mitochondrial autophagy and triggers mitochondrial apoptosis. A decline in mitochondrial function and number is also an important factor associated with a decrease in locomotor function [33]. Unfortunately, we did not measure this index in the chronic fatigue rat model due to sampling issues. The production and accumulation of free radicals during exercise are also one of the causes of exercise fatigue, and many studies have been conducted to promote fatigue recovery by supplementing exogenous antioxidants or antioxidant drugs to increase the activity of antioxidant enzymes [34]. In experimental animal models, astaxanthin supplementation demonstrated to enhance the activities of the endogenous antioxidant enzymes [35–36]. In this study, we examined the serum antioxidant enzyme activities in rats suffering from chronic fatigue, who had undergone one exhaustive exercise. We found serum GSH-Px activity was significantly increased in the AXE group compared with the E group. Furthermore, CAT activity and GSH/GSSG, although not significant (probably due to large individual differences), exhibited an increasing trend.

It is worth noting that although fatigue is related to the content and quality of skeletal muscle, this study found that astaxanthin alleviated chronic fatigue by enhancing the activity of antioxidant enzymes (such as GSH-Px and CAT), without further testing on skeletal muscle. Therefore, the effects of astaxanthin on myasthenia gravis remain unclear. Compared with other antioxidants (such as vitamin C and coenzyme Q10), astaxanthin’s strong lipophilicity allows it to specifically accumulate in mitochondrial membranes, potentially providing more significant targeted protective effects. Its specificity, however, requires further validation in future studies.

The sample size in this study was small (n=5 per group), which may reduce statistical power. Although the use of inbred Sprague-Dawley rats ensures a consistent genetic background, the limited sample size may still affect the applicability of the findings. Larger samples are needed in future studies to confirm these results.

## Conclusions

In the context of chronic exercise-induced fatigue, astaxanthin has been shown to enhance the antioxidant capacity of skeletal muscles, thereby improving mitochondrial function. In addition, astaxanthin has been demonstrated to possess anti-fatigue properties, contributing to the alleviation of fatigue symptoms.

## Figures and Tables

**Table 1:** Training program for rats with chronic exercise fatigue

Week	1–2 Weeks	3–4 Weeks	5–6 Weeks	7–8 Weeks
*Speed*	10 m/min	15 m/min	20 m/min	30 m/min
*Duration*	20 min/day	20 min/day	30 min/day	40 min/day
*Frequency*	5 days/week	5 days/week	5 days/week	5 days/week
*Incline*	0°	0°	5°	5°

**Table 2:** Grouping of rats with chronic exercise fatigue (n = 5)

Group	Treatment Protocol
*Control (Con, n=5)*	Daily oral gavage of soybean oil (1 ml/100 g body weight) + routine treadmill training.
*Astaxanthin (AX, n=5)*	Daily oral gavage of astaxanthin (3 mg/100 g body weight, dissolved in soybean oil) + routine treadmill training.
*Exhaustive Control (E, n=5)*	Routine training followed by a single bout of exhaustive exercise + soybean oil gavage.
*Astaxanthin Exhaustion (AXE, n=5)*	Routine training followed by a single bout of exhaustive exercise + astaxanthin gavage.

## Abbreviations

AX: Astaxanthin
AXE: Astaxanthin exhaustion exercise
CAT: Catalase
CK: Creatine kinase
GPX: Glutathione peroxidase
NEFA: Non-esterified fatty acid
BUN: Blood urea nitrogen
MMP: Mitochondrial membrane potential
